# The pre-hospital pulse: high responsiveness, deployment, and utilisation of ambulance services in India

**DOI:** 10.5588/pha.26.0028

**Published:** 2026-08-24

**Authors:** M. Moonis, V. Madhur, S. Yadvinder, K. Anil, S.A. Parray, P. Thekkur, K. Rakesh, K. Niraimathi, Z. Rony, A. Grover

**Affiliations:** 1 Department of Hospital Administration, All India Institute of Medical Sciences, Bathinda (Punjab), India;; 2 Department of Community and Family Medicine, All India Institute of Medical Sciences, Bathinda (Punjab), India;; 3 National Health Mission, Panchkula, Haryana;; 4 Director Health Services. ESI, Punjab, India;; 5 Special Secretary, Finance Department, Punjab (IAS), India;; 6 Centre for Operational Research, International Union against Tuberculosis and Lung Disease, Paris, France;; 7 Evidentia Research Solutions, Chennai, India;; 8 UNICEF, UNDP, World Bank, WHO Special Programme for Research and Training in Tropical Diseases (TDR), Geneva, Switzerland;; 9 Head Division of Implementation Research, Extramural Division, Indian Council of Medical Research, New Delhi, India.

**Keywords:** SORT IT, universal health coverage, Sustainable Development Goals, pre-hospital care

## Abstract

**BACKGROUND:**

India’s National Ambulance Service, a key component of the public health system, operates through 102, 108, and 112 hotline networks.

**OBJECTIVE:**

To assess ambulance performance in terms of responsiveness, deployment, and utilisation. A descriptive study was conducted using ambulance records from two large states in India, Punjab and Haryana, from January to December 2025.

**RESULTS:**

Of 626,372 hotline calls (72% from rural areas), 97% resulted in ambulance deployment, and 92% were utilised for patient transfers. 91% of ambulances reached the pickup location within 30 min (response time) and 67% transported patients to health facilities within 60 min (call-to-drop time). Median distance travelled by ambulance (parking to drop location) was 32 km (interquartile range 17–58). In Punjab, 50% of call-to-drop times were within 60 min, while in Haryana this was 74%. There were notable interdistrict variations in call-to-drop times. The most common reasons for hotline calls were maternal and obstetric conditions (37%), medical emergencies (27%), and trauma or accidents (17%). Inter-facility transfers accounted for 61% of ambulance utilisation.

**CONCLUSION:**

Our study demonstrates that ambulance services perform at a high level and provide substantial public health value. However, opportunities remain to further strengthen performance, particularly in reducing call-to-drop times.

## Graphical abstract

**Figure:**
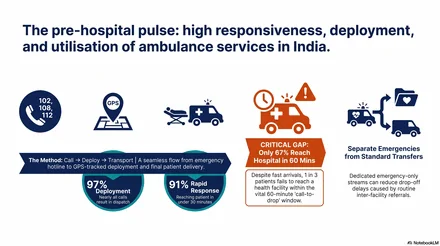

Emergency ambulance services are a critical component of pre-hospital care, enabling timely patient transport and linking communities to formal health facilities.^1,2^ India’s National Ambulance Service (NAS) operates through the 102, 108, and 112 hotline networks.^3–5^ As per national standards, one advanced life support ambulance is recommended for every 500,000 people and one basic life support ambulance for every 100,000 people.^6^ The desired ambulance response time (hotline call to arrival at scene) is 30 min. The maximum desired ‘call-to-drop time’ (hotline call to arrival at health facility) is 1 h.^7^ Evidence from low- and middle-income countries (LMICs) shows that rapid ambulance response time improves survival and reduces disability.^8^

Punjab and Haryana, two states located in northern India, face infrastructural and geographic challenges that may impact ambulance service performance. There are few studies evaluating the response time, utilisation patterns, and outcomes of government-run emergency ambulance services in these two states.^9–13^ These studies focused on specific aspects of ambulance activity and were limited geographically. None of these studies provided a comprehensive evaluation of ambulance performance including responsiveness, deployment, and utilisation on a large scale and over a full year that covers seasonal variations. Such information would be useful to inform operational and policy decisions on optimising ambulance performance in India and other resource-limited settings.

We therefore aimed to assess the performance of ‘Government-run’ emergency ambulance services in two large states (Punjab and Haryana) of India. The specific objectives were to: 1) determine the total number of hotline calls and the proportion that resulted in ambulance deployment and utilisation; 2) assess ambulance response time, patient transport duration, and the distance from the pickup to drop location; and 3) describe the demographic profile of patients, reasons for ambulance utilisation, and emergency care offered during ambulance transfer.

## METHODS

This was a longitudinal descriptive study using secondary programme data extracted from routine records of the government-run ambulance referral services between 1st January 2025 and 31st December 2025.

### General setting

India is the world’s most populous country and operates a tiered public health care delivery system with three levels: primary, secondary, and tertiary care. Primary care is delivered through community-based facilities such as sub-centres and primary health centres, which provide preventive, promotive, and basic curative services. Secondary care is delivered through community health centres, sub-district hospitals, and district hospitals that provide more specialised services. Tertiary care is provided through medical colleges and specialised referral hospitals. Ambulance services facilitate patient transfers across these three levels of care.^14,15^

### Specific Setting

The study was conducted in Punjab and Haryana, two neighbouring states in northern India with estimated populations of about 34 million and 32 million, respectively.^16^ Both states operate 24-h, free-of-charge ‘government-supported’ ambulance services staffed by trained emergency medical technicians and drivers. Emergency calls are received through centralised control rooms linked to national emergency telephone networks, including 102, 108, and 112.^4,17^ In Haryana, ambulance services are coordinated by the National Health Mission and delivered through 522 ambulances. In Punjab, ambulance services are managed by the Punjab Health Systems Corporation with 356 ambulances. Central control rooms receive calls, allocate ambulances, track vehicles through GPS-enabled systems, and maintain real-time dashboards that monitor response times and operational performance.^4,17^

### Recording and reporting system for ambulance operations

In Haryana, operational data are recorded through the Emergency Response Support System, while in Punjab the reporting system is integrated with the District Health Information Software platform. In both states, ambulances are equipped with Mobile Data Terminals (MDTs) that record dispatch information, vehicle movement, and call closure. GPS/GPRS-based tracking systems capture timestamps and route movements, enabling calculation of response time, transport time, and travel distance. When a hotline call is received, the nearest available ambulance is identified through the centralised control room and assigned to the case. Dispatch details are transmitted electronically to the ambulance through the MDT. After the assigned ambulance reaches the pickup location (deployed), the patient is loaded and transported to an appropriate receiving facility (utilised), typically the nearest government health institution, although patient preference may influence the choice of destination. Upon arrival at the receiving facility, the patient is handed over to the treating team and task completion is recorded electronically.

### Operational definitions

A ‘hotline call’ refers to any call received by the centralised control room on the 102, 108, or 112 emergency numbers. ‘Ambulance deployment’ is defined as a hotline call that resulted in the dispatch of an ambulance to the pickup location, as recorded electronically by the MDT. ‘Ambulance utilization’ is defined as a deployed trip in which a patient was loaded and transported to a receiving facility or other destination. ‘Response time’ is the interval from receipt of the hotline call to ambulance arrival at the pickup location; ‘Call-to-drop time’ is the interval from receipt of the hotline call to the patient’s arrival at the receiving health facility.

### Data source, variables, and collection

The primary data source for this study was the electronic ambulance network database maintained by the respective state governments. Data variables were extracted from these databases and included: state, district, date of hotline call, total number of ambulances, number of hotline calls, response time, transport time, call-to-drop time, distance travelled, reasons for hotline calls, pickup and drop locations, and type of emergency care provided. As the reasons for ambulance calls were recorded inconsistently across the two states, these were standardised by the study team into the following categories: maternal or obstetric conditions, neonatal or paediatric conditions, trauma or accident, medical emergencies, poisoning or bites, referral or inter-facility transfers, transport back-home, administrative and maintenance, and not recorded. Data quality assurance was undertaken regularly at the district level as part of the routine programme. Monitoring teams cross-checked digital records against physical logbooks and dispatch registers. They also reviewed operational indicators such as response time and fuel consumption and conducted occasional field verification of ambulance locations and operational status.

### Statistical analysis

Analysis was performed using Microsoft Excel. Categorical variables were summarised using frequencies and proportions. Continuous variables, including response time, transport time, call-to-drop time, and travel distance, were summarised using median and interquartile range (IQR). Using the desirable thresholds for response time (≤30 min) and call-to-drop time (≤60 min), we estimated proportions that crossed these thresholds in both states. Missing data were retained as a separate ‘not recorded’ category and are reported as such in all tables. Records with data entry errors were excluded from the respective variable-level analyses.

### Ethical statement

Ethics approval was received from the All India Institute of Medical Sciences, Bathinda (IEC/AIIMS/BTI/2025/11/31; dated 29-11-2025). Additionally, necessary permissions were sought from the Director, National Health Mission, Haryana, and the Director, Punjab Health Systems Corporation, for using ambulance service data required for this study. As the study involved anonymised programme data, the requirement for informed consent did not apply.

## RESULTS

### Hotline calls, ambulance deployment, and utilisation

Table 1 shows the number of hotline calls, ambulance deployment, and utilisation in Punjab and Haryana. Of 626,372 total calls received, 97% resulted in ambulance deployment and 92% were utilised for patient transport. The overall median response time was 10 min (IQR 6–27) and the median call-to-drop time was 47 min (IQR 43–87). Following a hotline call, 91% of ambulances arrived at scene in ≤30 min. Regarding call-to-drop time, 67% of ambulances transported patients to a health facility within 60 min. In Punjab, 50% of call-to-drop times were within 60 min, while in Haryana this was 74%. Figure 1 shows the proportion within the desired ambulance response time and call-to-drop times in Punjab and Haryana.

**TABLE 1.** Hotline calls, ambulance deployment, and utilisation in Punjab and Haryana, India (Jan–Dec 2025).

Variables	Punjab		Haryana		Total	
	n	%	n	%	n	%
Population^A^	33,927,403		32,205,519		66,132,922	
Ambulances available	356		522		878	
Hotline calls						
Total calls received	219,396	100	406,976	100	626,372	100
Ambulances deployed	215,995	98	392,136	96	608,131	97
Ambulances utilised	185,618	85	391,580	96	577,198	92
Average calls per ambulance per day	2		2		2	
Average calls per 100,000 population	2		4		3	
Response time minutes (median, interquartile range [IQR])	17	6–27	7	3–15	10	3–20
Response time (n %)						
≤30 min	175,127	83	369,466	95	544,593	91
31–60 min	35,692	16	15,189	4	50,881	8
>60 min	1369	1	4669	1	6038	1
Call-to-drop time minutes (median, IQR)	60	43–87	41	26–62	47	30–71
Call-to-drop time (n %)						
≤60 min	35,996	50	276,966	74	367,962	67
61–90 min	47,826	26	54,280	15	102,106	18
>90 min	42,659	24	44,133	12	86,792	16
Distance – ambulance station to drop location km (median, IQR)	28	16–48	35	12–62	32	17–58
Time of call						
12 AM to 6 AM	28,251	13	54,332	13	82,583	13
6 AM to 12 PM	56,586	27	128,222	33	184,808	31
12 PM to 6 PM	67,918	37	137,629	35	205,547	36
6 PM to 12 AM	66,641	30	86,793	21	153,434	25
Seasonal trends						
Jan–March	51,374	23	91,205	22	142,579	23
April–June	51,539	24	95,255	24	146,794	24
July–September	62,046	34	116,237	30	178,283	31
October–December	54,437	25	104,279	26	158,716	25

Note: All percentages are calculated using the total hotline calls received as the denominator.

A Projected population of Punjab and Haryana, 2026. https://www.iipsindia.ac.in/sites/default/files/FULL_REPORT_WITH_FINAL_TABLES.pdf.

**FIGURE 1.**
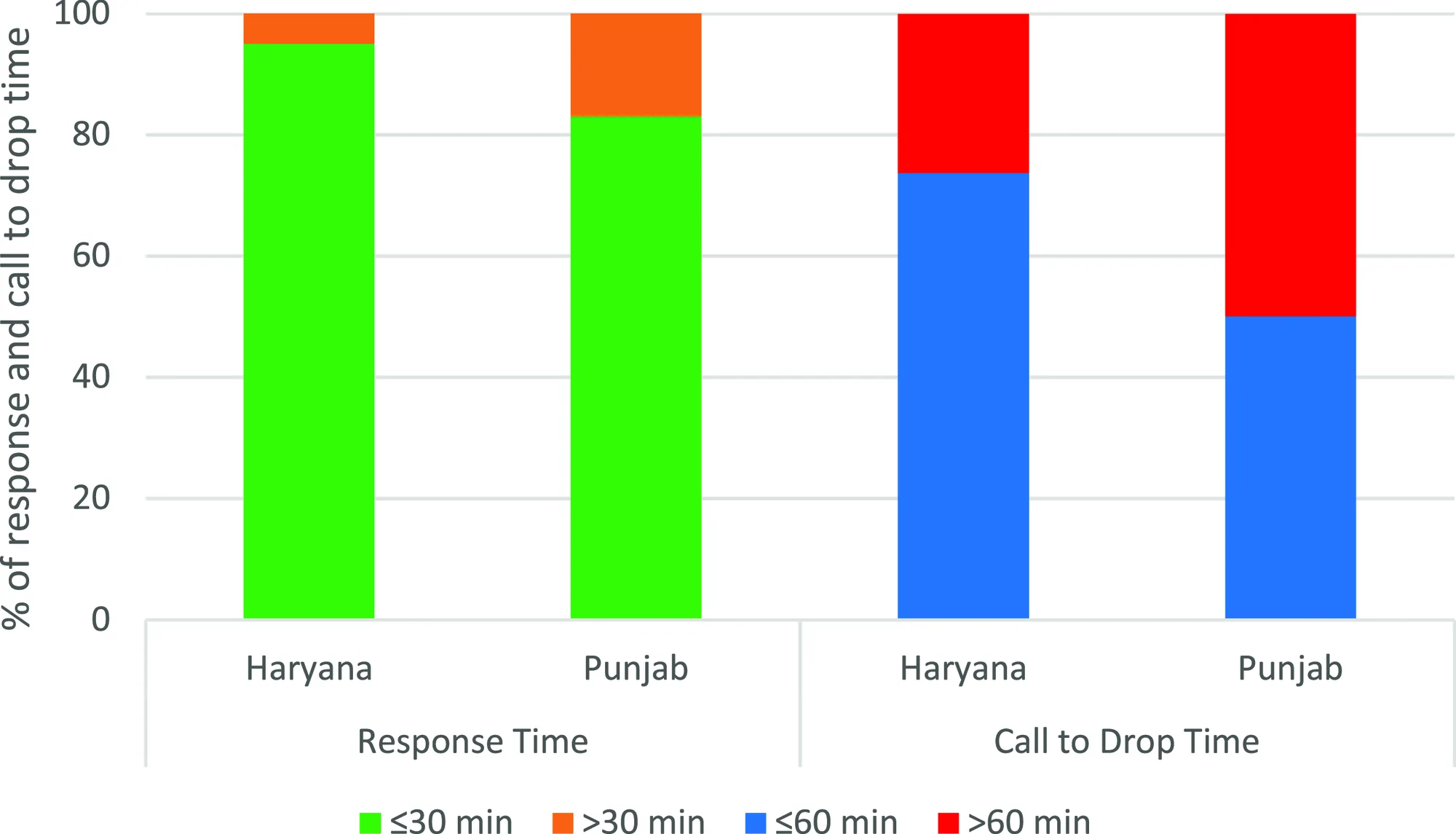
Ambulance response time and call-to-drop time in Punjab and Haryana, India (2025).

Figures 2 and 3 show notable interdistrict variations in proportions meeting the desired call-to-drop times. In Punjab, the districts where maximum proportion of patients were dropped off within 60 min were SAS Nagar (73%), Bathinda (68%), Patiala (59%), and Amritsar (58%). Similarly in Haryana, the highest proportions were seen in Rohtak (99%), Jhajjar (99%), Nuh (99%), and Panipat (92%).

**FIGURE 2.**
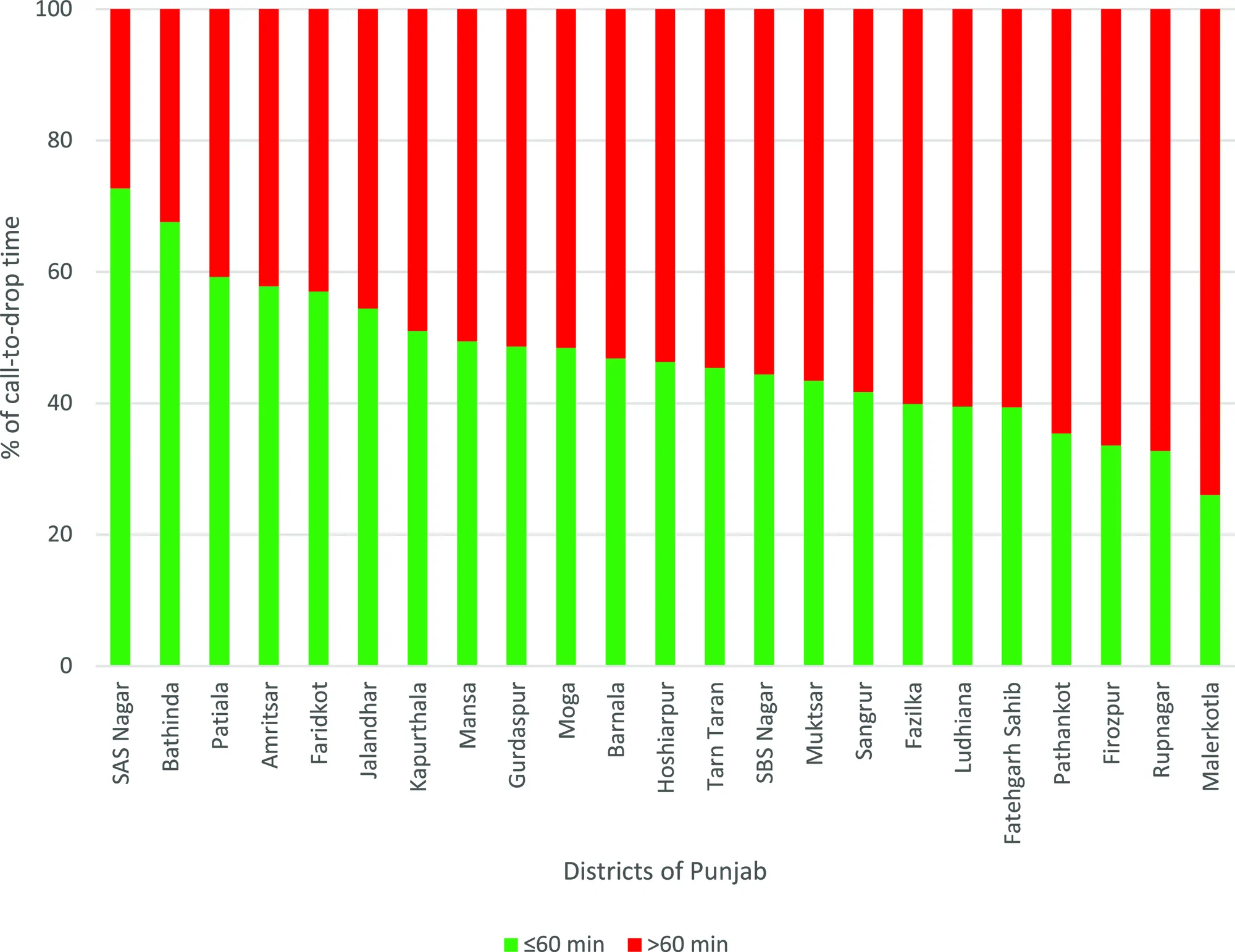
District-wise distribution of call-to-drop times in Punjab, India (2025).

**FIGURE 3.**
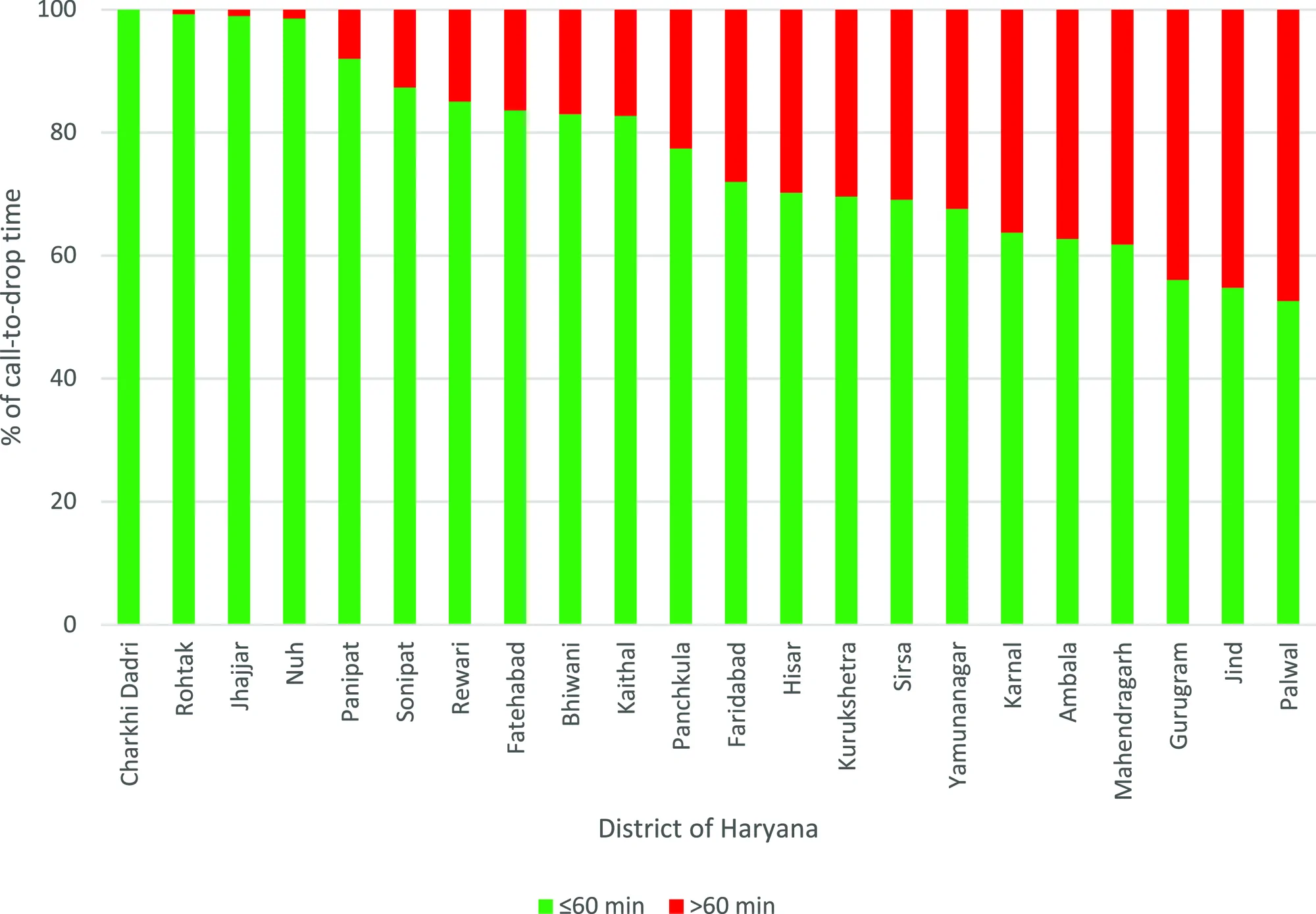
District-wise distribution of call-to-drop time in Haryana, India (2025).

### Reasons for ambulance calls, emergency care provided, and drop location

Of 626,372 cumulative calls, the majority (72%) originated from rural areas (Table 2). The three most common reasons for hotline calls were maternal and obstetric conditions (37%), medical emergencies (27%), and trauma and accidents (17%) (together constituting 81% of the reasons for calls).

**TABLE 2.** Demographic profile, reasons for ambulance utilisation, emergency care, and drop location in Punjab and Haryana, India (Jan–Dec 2025).

Variables	Punjab		Haryana		Total	
	n	%	n	%	n	%
Population	33,927,403		32,205,519		66,132,922	
Gender						
Women	96,710	44	272,522	67	369,232	59
Men	121,730	56	103,293	25	225,023	36
Not recorded	956	<0.1	31,161	8	32,117	5
Residence						
Rural	127,122	58	323,087	79	450,209	72
Urban	92,274	42	83,889	21	176,163	28
Age group						
<5 years	27,534	13	81,215	20	108,749	17
5–17 years	11,871	5	10,896	3	22,767	4
18–29 years	78,917	36	230,873	57	309,790	50
31–45 years	47,054	21	50,984	13	98,038	16
46–59 years	28,611	13	18,786	5	47,397	8
≥60 years	25,409	12	14,222	4	39,631	6
Reason for hotline calls						
Maternal and obstetric	51,687	24	177,243	44	228,930	37
Medical emergencies	89,591	41	81,883	20	171,490	27
Trauma and accident	61,433	28	45,635	11	107,069	17
Neonatal and paediatric	4,022	2	18,119	4	22,141	4
Transport back-home	18	<0.1	20,589	5	20,590	3
Referral and transfer	143	<0.1	11,503	3	11,646	2
Poisoning and bites	5,168	2	1,508	0	6,676	1
Administrative and maintenance	7,334	3	15,971	4	23,305	4
Not recorded	0	<0.1	34,525	8	34,526	6
Emergency care						
Cervical collar	Not recorded		745	<0.1	—	—
Cardiopulmonary resuscitation	Not recorded		1,472	<0.1	—	—
Automated external defibrillator	Not recorded		90	<0.1	—	—
Ventilator support	Not recorded		129	<0.1	—	—
Delivery conducted	Not recorded		55	<0.1	—	—
Death before ambulance arrival	904	<0.1	347	1	1,257	<0.1
Death during transport	Not recorded		6	<0.1	6	<0.1
Pickup and drop location						
Inter-facility transfer	102,424	47	280,923	69	383,347	61
Pickup (home/site to health facility)	116,954	53	91,726	23	280,697	33
Transport back-home	18	<0.1	32,154	8	32,155	5
Details not available	—	—	2,173	1	2,173	<0.1
Reason for inter-facility transfer						
Facility not available	78,360	77	Not recorded		—	—
Staff not available	24,064	24	Not recorded		—	—

Inter-facility transfers constituted 61% of ambulance utilisation. A total of 1,257 deaths were recorded prior to ambulance arrival at the scene, corresponding to a pre-arrival death rate of 201 per 100,000 hotline calls overall. A descriptive state-level comparison showed a higher rate in Punjab (412 per 100,000) than in Haryana (85 per 100,000).

In Haryana, 32,154 (8%) ambulance trips involved transporting individuals back to their homes following an emergency call.

## DISCUSSION

Drawing on over 600,000 hotline calls across a full calendar year, this study provides one of the most comprehensive evaluations of government-run emergency ambulance performance in India. The findings indicate that the vast majority of calls resulted in ambulance deployment (97%) and patient transport (92%), reflecting strong operational functioning of the system. The overall median response time was 10 min and call-to-drop time was 47 min. The majority of hotline calls originated from rural areas and 81% were for urgent medical conditions including maternal and obstetric cases, medical emergencies, and trauma or accidents.

These findings have important public health implications, underscoring the critical role of ambulance services as a backbone of emergency obstetric care and broader emergency referral systems in northern India.^18,19^ The high rates of ambulance deployment and utilisation indicate that the hotline call system is effective and widely used by the population. The study’s findings, particularly the high deployment and utilisation rates, offer transferable insights for low-resource settings seeking to strengthen emergency ambulance services. Strengthening emergency care systems has the potential to prevent more than half of avoidable deaths in LMICs.^20,21^

The study’s strengths include the use of a large dataset and full year of observations, enabling the assessment of seasonal patterns and supporting insights that may be broadly applicable to similar settings in India, though direct generalisation to states with different geography and infrastructure requires caution. It provides a comprehensive evaluation of real-time operational metrics (response and patient transfer times), patient characteristics, and reasons for ambulance utilisation. The study also addresses a recognised operational research priority and is therefore well positioned to inform policy and practice.^20,22^ Finally, we adhered to Strengthening the Reporting of Observational Studies in Epidemiology (STROBE) guidelines for the reporting of observational studies in epidemiology.^23^ One of the main limitations was the lack of standardised recording of reasons for hotline calls leading to ambulance deployment, which may have resulted in misclassification. To minimise this bias, two independent members of the study team classified the reasons, with discrepancies resolved through consensus. A potential way forward would be the introduction of a standardised reporting form within national and state monitoring and evaluation system frameworks. There were also some missing data on the reasons for hotline calls, the type of emergency care offered during ambulance transport, and reasons for inter-facility transfers. Strengthening training and feedback mechanisms for field teams would help improve the completeness and quality of documentation.^24^

The study findings have several implications for policy and practice. First, 50% of call-to-drop times exceeded 60 min in Punjab and 26% in Haryana, together with the wide interdistrict variation, suggesting infrastructural and geographic heterogeneity. Plausible contributors include differences in road connectivity, ambulance-to-population ratios, and district-level variation in health care facilities. In addition, the high volume of inter-facility transfers, accounting for 61% of all utilised ambulance trips, indicates that the emergency ambulance system is functioning predominantly as a referral-transport service rather than as a pre-hospital emergency-response service. Referral-system inefficiencies illustrated by the finding that in Punjab, 77% of inter-facility transfers were driven by ‘facility not available’ appear to be a systemic rather than sporadic driver of ambulance demand. Performance could be improved by emergency-response prioritisation, dedicated inter-facility transfer subset separated from an emergency-response subset, coordinated referrals,^15^ increasing ambulance availability, optimising pre-positioning relative to high-demand areas, and addressing operational constraints through structured performance reviews.^25^ However, the overall performance of the ambulance service in both states compares favourably with that reported from Kenya, South Africa, and other LMICs, where ambulance response times are often substantially longer and emergency medical coverage remains limited.^24,26,27^ Second, a concerning finding was that 1,257 deaths had occurred prior to ambulance arrival at the scene. As the aggregate operational dataset used here does not capture case severity at the time of the call, the help-seeking interval, or the patient’s clinical trajectory before ambulance arrival, any attribution to delayed help-seeking or to pre-existing clinical severity would therefore be speculative. A better understanding of these circumstances is needed before targeted information, education, and communication (IEC) strategies are designed.^18,24^ Finally, in Haryana, 32,154 ambulance trips were used for post-discharge transport. As ambulance services are primarily intended for emergency referrals, this observation warrants further review and consideration of potential resource optimisation.^18,19^

## CONCLUSION

Ambulance services in Punjab and Haryana demonstrate high performance, substantial public health value, and opportunities for further strengthening particularly in call-to-drop times. This type of operational research embedded within routine service delivery and using programmatic data can help make the ambulance system data rich, information rich, and action rich. It is both valuable and scalable and could be replicated in other settings across the country.
